# Impact of shade on outdoor thermal comfort—a seasonal field study in Tempe, Arizona

**DOI:** 10.1007/s00484-016-1172-5

**Published:** 2016-05-18

**Authors:** Ariane Middel, Nancy Selover, Björn Hagen, Nalini Chhetri

**Affiliations:** 1 School of Geographical Sciences and Urban Planning, Arizona State University, PO Box 875302, Tempe, AZ 85287-5302 USA; 2 School of Geographical Sciences and Urban Planning, State Climate Office, Arizona State University, Tempe, AZ 85287-1058 USA; 3 Julie Ann Wrigley Global Institute of Sustainability, Arizona State University, PO Box 878009, Tempe, AZ 85287-8009 USA

## Abstract

Shade plays an important role in designing pedestrian-friendly outdoor spaces in hot desert cities. This study investigates the impact of photovoltaic canopy shade and tree shade on thermal comfort through meteorological observations and field surveys at a pedestrian mall on Arizona State University’s Tempe campus. During the course of 1 year, on selected clear calm days representative of each season, we conducted hourly meteorological transects from 7:00 a.m. to 6:00 p.m. and surveyed 1284 people about their thermal perception, comfort, and preferences. Shade lowered thermal sensation votes by approximately 1 point on a semantic differential 9-point scale, increasing thermal comfort in all seasons except winter. Shade type (tree or solar canopy) did not significantly impact perceived comfort, suggesting that artificial and natural shades are equally efficient in hot dry climates. Globe temperature explained 51 % of the variance in thermal sensation votes and was the only statistically significant meteorological predictor. Important non-meteorological factors included adaptation, thermal comfort vote, thermal preference, gender, season, and time of day. A regression of subjective thermal sensation on physiological equivalent temperature yielded a neutral temperature of 28.6 °C. The acceptable comfort range was 19.1 °C–38.1 °C with a preferred temperature of 20.8 °C. Respondents exposed to above neutral temperature felt more comfortable if they had been in air-conditioning 5 min prior to the survey, indicating a lagged response to outdoor conditions. Our study highlights the importance of active solar access management in hot urban areas to reduce thermal stress.

**Electronic supplementary material:**

The online version of this article (doi:10.1007/s00484-016-1172-5) contains supplementary material, which is available to authorized users.

## Introduction

Outdoor thermal comfort is a complex function of atmospheric conditions and physical, physiological, psychological, and behavioral factors. These conditions and factors induce a subjective integrated response, thermal sensation, which has been the focus of many human biometeorology studies (Chen and Ng 2012; Johansson et al. 2014). Previous research has concentrated on identifying the factors that determine thermal comfort and breaking down their relative importance for thermal sensation using mixed methods that combine subjective and objective thermal assessments (e.g., Spagnolo and de Dear 2003; Eliasson et al. 2007; Kántor et al. 2012; Yin et al. 2012; Krüger et al. 2013; Pearlmutter et al. 2014). While indoor thermal comfort studies are usually conducted in climate-controlled conditions and can draw on several existing guidelines and standards (Johansson et al. 2014), the assessment of outdoor thermal comfort in cities is more challenging, as thermal conditions are less stable. Urban areas are heterogeneous and encompass various urban forms (type, density, and arrangement of buildings), surface materials, and landscapes, creating local scale and microscale climates that vary widely across space and time (Erell et al. 2012; Stewart and Oke 2012; Middel et al. 2014). Several studies have investigated thermal comfort in the context of urban form, focusing on street canyons or sky view factor (Johansson and Emmanuel 2006; Ali-Toudert and Mayer 2007; Pearlmutter et al. 2007; Mayer et al. 2008; Lin et al. 2010; Holst and Mayer 2011; Lee et al. 2014). Although the relationship between thermal comfort and the built environment tends to be strong, environmental factors, including meteorological conditions, generally only account for half of the variance in thermal sensation (Nikolopoulou et al. 2001; Nikolopoulou and Steemers 2003). The other 50 % can be attributed to a dynamic human parameter, which is composed of personal characteristics, i.e., age and gender; physiological factors such as weight and fitness level; psychological factors that include past experience, expectations, adaptation, thermal history, perceived control, and esthetic appreciation; and behavioral aspects such as clothing insulation, metabolic rate, time of exposure, and choice of location (e.g., Nikolopoulou and Lykoudis 2006; Vanos et al. 2010; Chen and Ng 2012; Klemm et al. 2015). All of these factors must be addressed in order to fully understand the integrated subjective thermal sensation response.

This study aims to quantify the impact of shade on subjective thermal sensation in a hot desert city—Tempe, Arizona—using subjective and objective comfort measures to address the environmental and non-environmental factors that impact thermal comfort. The importance of shade for reducing thermal stress in hot climates has already been emphasized by several authors (Johansson and Emmanuel 2006, Lin et al. 2010; Vanos et al. 2016). Our study objective is threefold: (1) examine the impact of shade on thermal comfort, perception, and perceived temperature; (2) investigate the relationship between atmospheric conditions and subjective thermal sensation; and (3) identify the most important drivers of outdoor thermal comfort in hot dry environments.

## Methodology

To quantify the thermal benefits of shade and investigate the relationship between perceived comfort and meteorological conditions outdoors, we conducted an objective and subjective assessment of thermal conditions through seasonal on-site meteorological observations and concurrent field surveys in Tempe, Arizona. Our assessment included sun-exposed locations as well as artificially shaded and tree shaded sites. We performed *t* tests to compare seasonal subjective thermal sensation in shaded and non-shaded locations and analyzed people’s air temperature estimates. Through regression analysis, we determined the physical drivers of thermal comfort. In a subsequent factorial ANCOVA, we examined how subjective thermal sensation varies by non-climatic factors after controlling for meteorological conditions. We then calculated physiological equivalent temperature (PET) from field observations and survey responses to determine neutral temperature, acceptable comfort range, and preferred temperature. Finally, we investigated the impact of air-conditioning on subjective thermal stress during pre-monsoon summer.

### Study site

**Fig 1:**
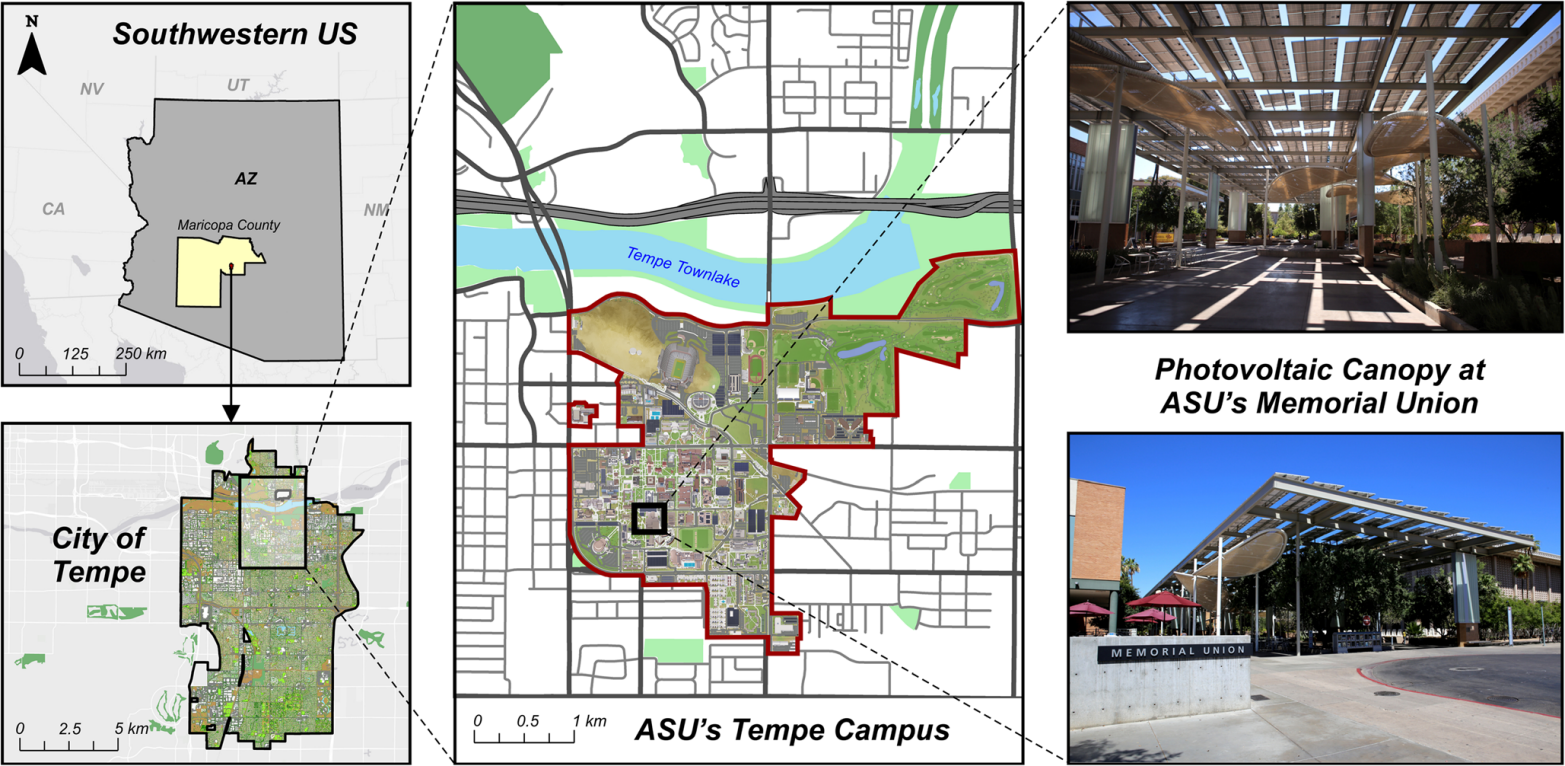
(From *left* to *right*) Geographic location of the City of Tempe in Maricopa County, Arizona, USA; Arizona State University’s Tempe Campus; Solar canopy structures provide shade at the Memorial Union on campus

The city of Tempe is located at 33.4294° N, 111.9431° W, 360 m above sea level, in the East Valley region of the Phoenix metropolitan area in Maricopa County, Arizona, USA (Fig. 1). The city encompasses a total area of 104.1 km^2^ and has a population of 172,816 (Census 2015), with increasing density in north Tempe, including the downtown area, and lower density development patterns in the south. According to the local climate zone (LCZ) scheme (Stewart and Oke 2012), Tempe downtown can be classified as mostly open and partly compact midrise to high-rise LCZ, while the rest of Tempe is mainly open lowrise. Situated in the Sonoran desert, Tempe has a semi-arid climate and a mean annual rainfall of 237 mm, most of it occurs during monsoon season in July and August (62 mm) and in the winter (December through March, 112 mm). June is extremely dry with less than 1 mm mean annual precipitation. Average maximum air temperature ranges from 39.3 to 40.4 °C between June and August to 20.1 to 22.6 °C between December and February. Mean minimum air temperature peaks at 24.0 °C in July and gets as low as 3 °C in December (WRCC 2015).

Downtown Tempe is home to the main campus of Arizona State University (ASU), a public university spread across four campuses in the Phoenix metropolitan area. ASU’s Tempe campus is about 2.6 km^2^, consists of broad pedestrian malls, and can be classified as open midrise LCZ. The Memorial Union, located in the heart of the Tempe campus, serves as community center for the ASU population and is a place of social interaction and gathering spot. The Memorial Union building offers student support amenities, restaurants, and services more than 14,000 people every day during the semester. The north and west exits lead to an expansive paved pedestrian mall, a walk-only zone from 8:00 to 16:00 h that used to have little vegetation, few mature trees, and little shade. In 2013, ASU partnered with a local utility provider and a solar energy company to cover the mall with three solar canopy structures to transform the open space. The installation was completed in May 2014, utilizing 1380 photovoltaic solar panels to cover 3330 m^2^ of land. The canopy structure now produces 397 kW DC and shades most of the pedestrian mall in front of the Memorial Union, including an outdoor dining area and a stage for outdoor events.

### Experimental design and meteorological measurements

In June 2014, we installed six shielded LASCAR Electronics EL-USB-2+ air temperature and relative humidity sensors under and near the solar canopy structures at the Memorial Union to monitor meteorological conditions at 5-min intervals for a full year (Fig. 1, supplemental materials). The sensors were mounted at 2.6-m height to prevent vandalism. Two of the sensors were attached to poles underneath the solar structure, 3.5 m below the photovoltaic panels over concrete. Two sensors were installed 30-m east and west of the structure at a lamp post over a grass patch (west) and over concrete pavement (east). The other two sensors monitor air temperature and relative humidity under dense tree canopies 30-m southeast of the structure over concrete pavement and 60-m southwest of the structure over grass. On clear and calm days representative of each season, we performed transects to collect additional data under each sensor. We chose June 10, 12, and 19, 2014 during pre-monsoon summer, November 7, 2014 in the fall, January 22, 2015 in the winter and April 2, 2015 in the spring. On those days, the average daily mean and maximum temperatures were less than 3 °C different from the seasonal normal (Table 1, supplemental materials). Transects were conducted hourly between 7:00 and 18:00 h local time and took about 20 min. We measured air temperature, relative humidity, wind speed, globe temperature, dew point, and Wet Bulb Globe Temperature (WBGT) with a Kestrel 4400 Heat Stress Meter at 1.1-m height, which is the center of gravity of the human body for standing subjects (ISO 7726 1998). We observed incoming (K↓) and outgoing (K↑) shortwave radiation using a Matrix Mk 1-G Pyranometer and took surface temperature measurements below the stationary sensors with a DeltaTRAK 15002 infrared thermometer. All instruments comply with ISO 7726 (1998) standards for sensor measurement range and accuracy (Table 1).

**Table 1:** Sensor specifications and measurement height for stationary and handheld observations

Sensor	Variable(s)	Range	Accuracy	Height
LASCAR Electronics EL-USB-2+ (shielded)	Air temperature	−35° to +80 °C	± 0.3 °C	2.6 m
	Relative humidity	0 to 100 % RH	± 2.0 % RH	
Kestrel 4400	Air temperature	−10° to +55 °C	± 0.5 °C	1.1 m
	Relative humidity	0 to 100 % RH	± 3.0 % RH	
	Globe temperature	−10° to +55 °C	± 1.4 °C	
	WBGT	See temperature	± 0.7 °C	
	Wind speed	0.6 to 60.0 ms^−1^	Larger of 3 % of reading, least significant digit or 20 ft/min	
DeltaTRAK 15002	Surface temperature	−40° to 510 °C	± 2.0 °C	1.1 m
Matrix Mk 1-G Pyranometer	Solar radiation (incoming and outgoing shortwave)	0.35 to 1.15 μm	± 5 %	0.6 m

### Field survey design

Concurrent with the seasonal meteorological measurements in June, November, January, and April, we conducted questionnaire surveys under and near the photovoltaic canopies between 7:00 and 18:00 h. The surveys were designed to be transversal, i.e., each respondent only participated once, and could be completed in 3–5 min. Although the surveys were administered randomly, the set-up was quasi-experimental, because the respondents were mainly ASU students, staff, and faculty. The questionnaire covered personal characteristics, psychological and environmental factors, contextual information, and self-reported thermal perception, affective evaluation of comfort, and preference. First, respondents were asked to disclose their health-related mood on a 5-point scale: *very bad* (0), *bad* (1), *fair* (2), *good* (3), or *very good* (4). To assess the level of physical and cultural thermal adaptation, we collected information on the time of residency in Arizona. Adaptation was coded into 4 climate familiarity categories: just moved here (*not familiar*), have experienced a summer in the desert before (*somewhat familiar*), have lived here for 5 years (*familiar*), have lived here for >5 years or moved here from another hot dry environment (*very familiar*). Subjects indicated the reason for being at the Memorial Union (*passing by*, *attending a class*, *meeting someone*, *lunch*/*resting*) as a measure of perceived control. To survey thermal perception, we collected subjective thermal sensation votes (TSV) on a semantic differential 9-point scale, which is particularly suitable for extreme environments: *very cold* (−4), *cold* (−3), *cool* (−2), *slightly cool* (−1), *neutral* (0), *slightly warm* (+1), *warm* (+2), *hot* (+3), and *very hot* (+4). Perceived comfort was evaluated on a 4-point scale from *comfortable* (0) to *very uncomfortable* (3). Subjects rated their thermal preference on a 7-point scale, ranging from *much cooler* (−3) to *neither warmer nor cooler* (0) to *much warmer* (+3). All subjective judgment scales we employed comply with ISO 10551 (1995). The last part of the survey requested personal characteristics (gender and age group), clothing information, and details about the respondents’ activity level and location 5 and 30 min prior to the survey (short-term and long-term thermal history). Respondents were also asked to estimate the current air temperature in the sun and in the shade. Finally, subjects noted their sun exposure (*full sun*, *shaded by the solar canopy*, or *shaded by a tree*) and the time of survey completion so that the responses could be linked to meteorological observations.

### Data processing

We calculated mean radiant temperature *T*_*mrt*_ [°C] from observed globe temperature *T*_*g*_ [°C], air temperature *T*_*a*_ [°C], and wind speed *V*_*a*_ [ms^−1^] for all transect locations and seasons using the following:

$$ {T}_{mrt}={\left[{\left({T}_g+273.15\right)}^4+\frac{1.1\cdot {10}^8{V}_a^{0.6}\ }{\left(\varepsilon {D}^{0.4}\right)\left({T}_g-{T}_a\right)}\right]}^{0.25}-273.15 $$

with globe emissivity *ε* = 0.95, globe diameter *D* = 0.0254 m, and the globe’s mean convection coefficient 1.1∙10^8^ *V*_a_^0.6^[ms^-1^] (Thorsson et al. 2007). Each survey response was linked to observed meteorological conditions and *T*_*mrt*_ either in full sun, under the solar structure, or in tree shade based on location, time, and date of the response. The self-reported short-term and long-term thermal history was recoded into binary variables indicating if the subject was exposed to air-conditioning (AC) 5 and 30 min before taking the survey. We converted clothing responses to clothing insulation units (clo) according to ISO 9920 (2007) and calculated the metabolic rates in Wm^−2^ (ISO 8996 2004) based on reported activities. In order to compare subjective thermal sensations to actual measured thermal conditions, we chose PET as biometeorological index (Mayer and Höppe 1987). PET has been widely used in outdoor conditions and allows us to compare our results to other thermal comfort studies (Johansson and Emmanuel 2006; Lin 2009; Hwang et al. 2011; Chen and Ng 2012; Kántor et al. 2012; Makaremi et al. 2012). We calculated individual PET values for each subject from meteorological observations, *T*_*mrt*_, clothing level, metabolic rate, and personal information using the MEMI model (Höppe 1999) implemented in Rayman (Matzarakis et al. 2007, 2010).

## Results

Figure 2 in the supplemental materials illustrates daily minimum, maximum, and mean air temperature and average relative humidity recorded by the stationary reference sensors at the Memorial Union between June 1, 2014 and May 31, 2015. The recorded sun-exposed shielded maximum air temperature was up to 2 °C higher than maximum air temperature in the shade. This relationship is reversed at night, with warmer minimum air temperature under the solar canopy and under trees, indicating a slight heat retention (up to 1 °C). The weather conditions during the selected field work days were clear and calm. Wind speed was low, averaging 0.6 ms^−1^ in the summer, 0.3 ms^−1^ in the fall, 1.1 ms^−1^ in the winter, and 0.5 ms^−1^ in the spring (Table 2, supplemental materials). On field work days in June 2014, air temperature reached 43.0 °C and globe temperature peaked at 51.7 °C in the sun, while relative humidity (water vapor pressure) was as low as 11.0 % (7.9 hPa). Weather conditions on November 7, 2014 (fall), January 22, 2015 (winter), and April 2, 2015 (spring) were milder, with maximum air temperature of 30.8, 19.3, and 30.6 °C; maximum globe temperature of 44.7, 32.8, and 43.3 °C; and an average daytime relative humidity (water vapor pressure) of 20.0, 15.3, and 16 % (8.5, 7.0, and 7.5 hPa).

We collected around 300 questionnaires in each season, yielding a total of 1284 valid samples (Table 2). Because of the quasi-experimental setup, sampling is biased towards the ASU student body. More than 80 % of the respondents fall into the 18–24 and 25–34 age groups. 58.8 % of the respondents are male, 41.2 % are female. Especially in the summer, sampling is biased towards shade; only few people (17.6 %) agreed to take the survey in full sun, because thermal conditions were stressful. Overall, respondents were in good health, only 0.4 to 4.3 % reported they felt bad or very bad (Table 3). The number of participants not familiar with the Arizona climate is higher in the summer than in all other seasons (20.9 vs. 11.5 % and less), because a lot of prospective out-of-state students were visiting ASU. Also, the main purpose to be at the Memorial Union in June was to attend summer school (36.6 %), while respondents were primarily passing by in the fall (42.3 %) or had lunch/rested in the winter (50 %) and spring (41.7 %). Table 3 provides a complete list of contextual and personal factors, including frequency distributions and mean responses.

**Table 2:** Personal characteristics of survey participants

Demographic variables		Summer (*N* = 306)	Fall (*N* = 364)	Winter (*N* = 338)	Spring (*N* = 276)
		[%]	[%]	[%]	[%]
Age Group	18–24	62.1	66.8	79.6	76.4
	25–34	19.9	18.4	16.6	13.4
	35–44	5.9	3.8	1.8	4.0
	45–54	4.6	4.9	1.8	2.9
	55–64	5.6	4.7	0.3	2.5
	65+	2.0	1.4	0.0	0.7
Gender	Male	60.1	61.5	55.9	57.6
	Female	39.9	38.5	44.1	42.4

**Table 3:** Contextual and personal factors covered in the survey; frequency distribution of survey responses for nominal and ordinal variables, mean responses for interval variables

Variable	Response	Summer (*N* = 306)	Fall (*N* = 364)	Winter (*N* = 338)	Spring (*N* = 276)
		[%]	[%]	[%]	[%]
Health-related mood	Very good	25.8	39.8	29.6	39.9
	Good	54.9	44.2	52.7	48.2
	Fair	15.0	14.3	13.9	11.6
	Bad	3.6	1.1	3.0	0.4
	Very bad	0.7	0.5	0.9	0.0
Climate adaptation	Very familiar	36.3	37.1	44.4	46.4
	Familiar	26.1	24.7	26.9	22.1
	Somewhat familiar	16.7	26.6	20.7	22.5
	Not familiar	20.9	11.5	8.0	9.1
Location choice	Passing by	22.9	42.3	19.8	26.1
	Class at Memorial Union	36.6	10.4	8.9	9.8
	Meeting someone	20.9	18.7	21.3	22.5
	Lunch/resting	19.0	28.6	50.0	41.7
Thermal perception (thermal sensation vote)	Very cold	0.0	0.5	3.0	0.0
	Cold	0.0	0.5	18.3	0.0
	Cool	1.6	10.7	26.6	7.6
	Slightly cool	1.6	20.1	32.5	15.9
	Neutral	6.9	27.2	9.2	26.8
	Slightly warm	19.6	24.2	6.2	26.1
	Warm	24.8	12.4	3.6	17.8
	Hot	25.2	3.3	0.6	5.4
	Very hot	20.3	1.1	0.0	0.4
Thermal comfort	Comfortable	38.6	67.9	54.7	75.0
	Slightly uncomfortable	40.2	19.2	35.8	18.5
	Uncomfortable	17.6	3.3	3.3	0.4
	Very uncomfortable	3.6	9.6	6.2	6.2
Thermal preference	Much cooler	10.8	1.9	1.8	4.3
	Cooler	26.1	9.1	2.7	11.2
	Slightly cooler	37.3	26.6	13.0	39.9
	Neither warmer nor Cooler	20.6	49.5	34.3	39.1
	Slightly warmer	2.0	10.2	35.2	3.3
	Warmer	2.0	2.5	11.2	1.4
	Much warmer	1.3	0.3	1.8	0.7
Sun exposure	Full sun	17.6	25.8	33.4	18.5
	Shaded (solar structure)	70.3	63.2	59.8	75.4
	Shaded (tree)	12.1	11.0	6.8	6.2
Short-term thermal history	No AC (5 min ago)	55.2	60.2	67.2	60.9
	AC (5 min ago)	44.8	39.8	32.8	39.1
Long-term thermal history	No AC (30 min ago)	27.1	27.5	30.2	22.8
	AC (30 min ago)	72.9	72.5	69.8	77.2
		Mean	Mean	Mean	Mean
Activity level	Metabolic rate (5 min ago)	101	110	103	104
	Metabolic rate (30 min ago)	90	90	89	86
Clothing	Clothing insulation	0.46	0.62	0.83	0.49
Air temperature estimate	Shade	34.3	23.7	16.2	25.3
	Sun	40.1	27.7	20.1	29.4

### Impact of shade on thermal comfort

To assess how shade impacts subjective thermal sensation, we investigated TSV for shaded and sun-exposed responses by season. First, we tested for differences in TSV between shade types (artificial vs. natural shade), comparing TSV of subjects in tree shade to solar canopy shaded responses. An independent samples *t* test revealed no significant differences in seasonal TSV reported under the photovoltaic canopy and under trees. The effect for shade type was not significant in any season (Table 4a), indicating that natural (tree) and artificial (photovoltaic) shade have the same effect on thermal perception. Therefore, subsequent analyses will not differentiate between shade types and only investigate shaded vs. sun-exposed responses.

**Table 4:** (a) Independent samples *t* test for thermal sensation votes of artificially and naturally shaded respondents. (b) Independent samples *t* test for thermal sensation votes of shaded and sun-exposed respondents (**p < .001). We assume that the semantic differential 9-point TSV scale has interval properties, meaning that distances between points on the scale are equal. A non-parametric independent samples Mann-Whitney *U* test confirmed the *t* test results

a) TSV	Photovoltaic shade		Tree shade		*t*	*df*	*p* (2-tailed)	Mean diff.
	Mean	*σ*	Mean	*σ*				
Summer	2.00	1.32	2.41	1.52	−1.71	250	0.890	−0.41
Fall	−0.13	1.27	0.03	1.35	−0.72	265	0.477	−0.16
Winter	−1.62	1.10	−1.48	1.53	−0.58	222	0.565	−0.14
Spring	0.26	1.36	0.82	1.07	−1.65	223	0.100	−0.56
b) TSV	Shade		Sun		*t*	*df*	*p* (2-tailed)	Mean diff.
	Mean	*σ*	Mean	*σ*				
Summer	2.06	1.35	2.93	1.29	4.33	304	0.000**	0.87
Fall	−0.11	1.29	0.99	1.40	6.94	358	0.000**	1.10
Winter	−1.61	1.13	−0.90	1.66	4.58	335	0.000**	0.70
Spring	0.31	1.35	1.26	0.89	4.80	274	0.000**	0.95

**Fig. 2:**
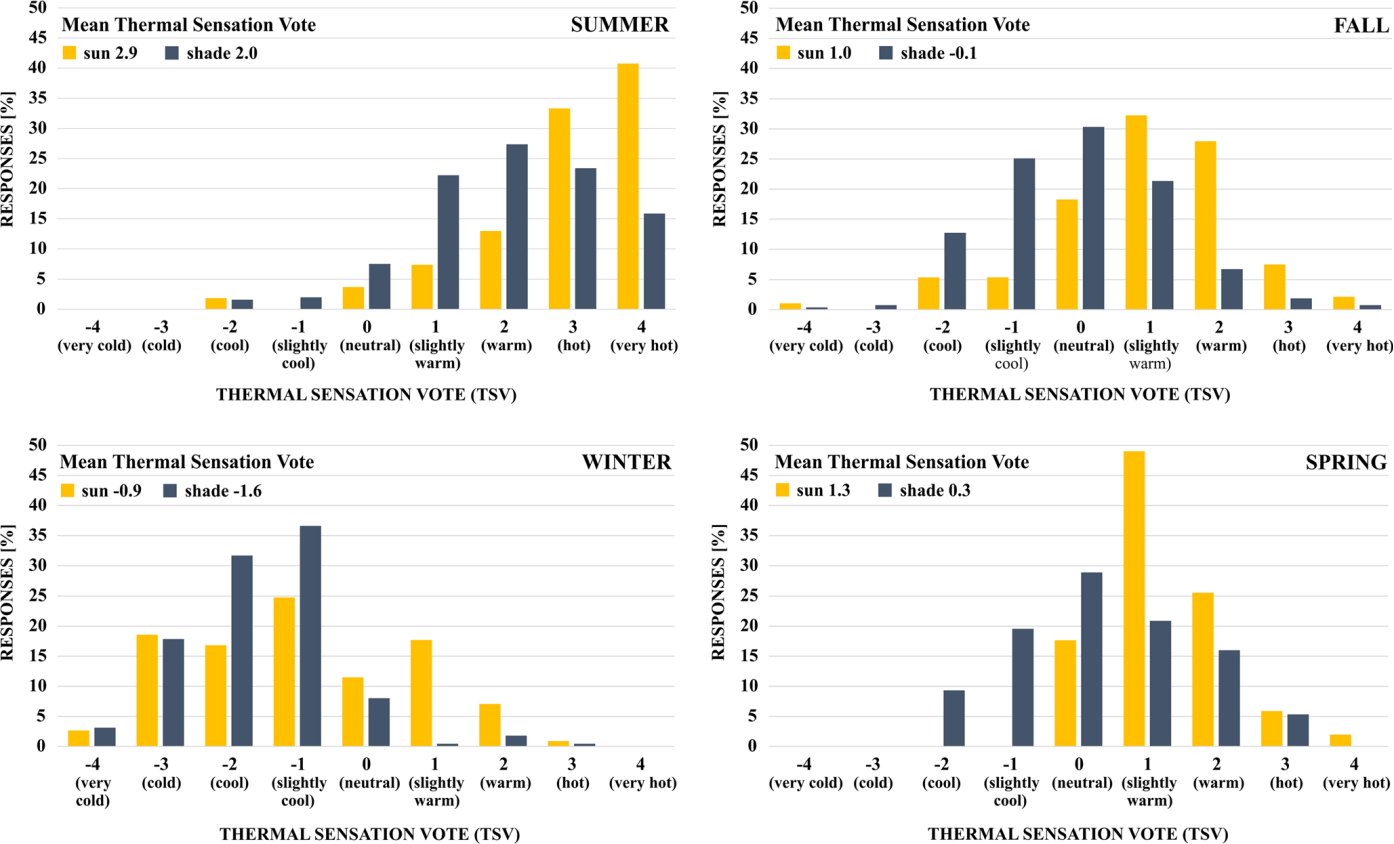
Frequency distribution (in percent) of subjective seasonal thermal sensation votes (TSV) for shaded and sun-exposed survey samples; seasonal mean thermal sensation votes (MTSV), sun vs. shade

Figure 2 illustrates the frequency distribution of TSV in the sun and in the shade, grouped by season. In the summer, 50.0 % of the subjects felt *warm* to *hot*, 20.3 % felt *very hot*, while only 3.2 % felt (*slightly*) *cool*. Responses during the transitional fall and spring seasons cluster around *neutral* TSV. In the winter, most subjects felt *slightly cool* to *cold* (77.4 %). Comparing sun-exposed and shaded responses, shade decreased perceived comfort by approximately 1 point on the 9-point scale for all seasons. Differences in TSV are statistically significant (Table 4b). Results show that shade relieves heat stress during the summer, lowering TSV from *hot* to *warm*. In transitional periods, shade improves perceived comfort from *slightly warm* to *neutral*, but conditions become more uncomfortable in the winter, with TSV changing from *slightly cool* to *cool*. These findings are in line with previous studies in hot humid climates. Lin et al. (2010) found that less open sites with decreased sky view factor improve thermal comfort in the summer but decrease comfort in the winter. Hwang et al. (2011) highlighted benefits of shade in the spring, summer, and fall but found that sun exposure improves thermal comfort in the winter.

**Fig. 3:**
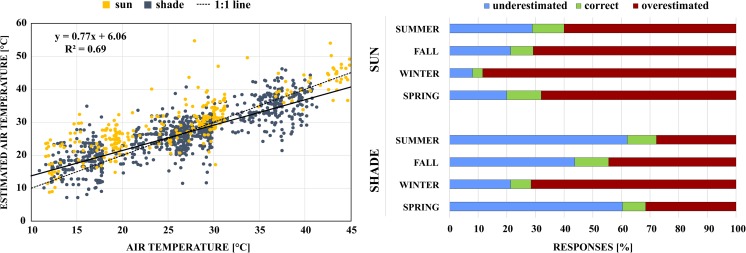
Observed air temperature (1.1 m height) vs. estimated air temperature for all samples (*N* = 1284) (*left*). Air temperature estimation error for shaded and sun-exposed survey samples by season (*right*)

We asked subjects to estimate air temperature in the shade and sun at the time of survey completion to assess how participants perceived ambient temperature. A regression of estimated on observed air temperature shows that overall, respondents underestimated warm and overestimated cool air temperature, with a threshold of 26.3 °C. Kántor et al. (2012) found the same trend for Szeged, Hungary with a threshold air temperature of 21.5 °C, while respondents in Göteborg, Sweden were very aware of the weather, only overestimating air temperature slightly (Thorsson et al. 2004). We further investigated estimation errors by season in the context of sun-exposure, dividing shaded and unshaded responses into three categories: overestimated air temperature, correct (estimate within ±0.5 °C of the actual air temperature), and underestimated air temperature (Fig. 3). We found that the majority of sun-exposed subjects (60–80 %) overestimated air temperature regardless of season. In contrast, respondents in the shade mostly underestimated actual air temperature in all seasons except winter. These findings indicate that solar access is an important driver of subjective outdoor thermal comfort.

### Drivers of thermal comfort

Previous outdoor thermal comfort literature has shown that thermal comfort is influenced by physical, psychological, physiological, and behavioral factors (Chen and Ng 2012). We investigated which factors are the most significant drivers of subjective thermal sensation, using meteorological observations and survey responses as independent variables. First, we used multiple regression analysis to identify the meteorological drivers for variations in TSV. Independent variables to explain TSV included observed air temperature, water vapor pressure (derived from relative humidity and air temperature), surface temperature, incoming and outgoing shortwave radiation, WBGT, and globe temperature. The linear combination of meteorological variables was significantly related to TSV, *F*(7,1271) = 218.64, *p* < 0.0001, with *R* ^2^ = 0.55. Globe temperature was the only significant predictor of TSV in the regression of observed meteorological variables, emphasizing the importance of the radiative environment for outdoor thermal comfort in hot and dry climates (Table 3, supplemental materials). In a separate regression between globe temperature and TSV, globe temperature explained 51 % of the variance in subjective thermal sensation, *F*(1,1277) = 1353.62, *p* < 0.0001 (Fig. 3, supplemental materials). These results are in agreement with previous studies that found a better correlation of thermal sensation with globe temperature than air temperature in Europe (Nikolopoulou and Lykoudis 2006) and a stronger effect of MRT on thermal comfort than air temperature in Malaysia (Makaremi et al. 2012). MRT, which can be derived from globe temperature or measured with three-dimensional short- and long-wave radiation sensors, has been identified as the most important variable for outdoor thermal comfort by various authors (e.g., Ali-Toudert and Mayer 2007; Mayer et al. 2008; Lee et al. 2013; Lee et al. 2014). Our results strongly suggest that, in hot and dry climates, solar access is more important for thermal comfort than humidity, as humidity levels are usually low (except during monsoon season). Therefore, measures such as WBGT and Heat Index (HI) are less suitable to predict thermal discomfort and heat stress in regions with low humidity levels, such as Arizona.

In a second step, we performed a factorial ANCOVA to include non-meteorological categorical factors in the analysis. Controlling for observed globe temperature as covariate, we compared the main effects of various survey responses on TSV and their interactions. To reduce the number of factors in the ANCOVA, we only included interaction terms that were significant in separate ANCOVAs (Table 5). The model revealed a significant effect of most of the non-meteorological variables on TSV after controlling for globe temperature (*R* ^2^ = 0.64**,** adjusted *R* ^2^ = 0.61). In accordance with Pantavou et al. (2013); Pearlmutter et al. (2014), and Lee et al. (2016), our results confirm that season and time of day significantly impact thermal comfort. The interaction terms of season and time of day with globe temperature had a mild effect on TSV. Thermal adaptation was also significant as main effect (*p* = 0.011) and as interaction term with sun exposure (*p* = 0.019), exhibiting a general trend of more adapted subjects reporting lower TSV. Long-term Arizona residents seem to be more adapted to the summer heat but feel cold more easily in the winter. The relevance of thermal adaptation and climatic region of origin for comfort was pointed out by Makaremi et al. (2012) who concluded that international students at University of Putra Malaysia felt less comfortable in outdoor conditions than local students. Similarly, on Caribbean beaches, tourists from tropical regions perceived conditions to be cooler than respondents from more temperate climates (Rutty and Scott 2015). Our ANCOVA did not reveal a significant effect of perceived control through choice of location on subjective thermal sensation (*p* = 0.531), as found by Nikolopoulou and Lykoudis (2006) and Pantavou et al. (2013). Also, health-related mood was not significant (*p* = 0.924), probably because the majority of respondents were healthy and the sample size of subjects who felt bad or sick was <5 %. Reported thermal comfort votes, their interaction with globe temperature and sun exposure, as well as thermal preferences contributed significantly to the variation in TSV, emphasizing the importance of personal experience and expectations (Nikolopoulou and Steemers 2003). Sun exposure was not significant in this year-round analysis (*p* = 0.294), but the interaction term of season and sun-exposure was (*p* = 0.011). In agreement with a study in hot dry Damascus (Yahia and Johansson 2013), previous activities of the respondents did not significantly influence subjective thermal comfort, as survey participants did not report a wide range of activities; respondents were mainly standing, walking, or sitting (also compare average metabolic rates in Table 2). The same rationale applies to clothing insulation. Due to mild winters in Arizona, t-shirts can be worn year-round and clothing insulation did not vary much amongst survey responses (*M* = 0.61, *SD* = 0.23). Pertaining to personal characteristics, age was not found to be significant (*p* = 0.884), but gender was (*p* = 0.004), with females reporting slightly lower TSV than males. Thermal history was not an important driver of subjective thermal sensation in this year-round analysis but will be analyzed in more detail for the summer.

**Table 5:** Results for a factorial ANCOVA with TSV as dependent variable and globe temperature as covariate; factors are categorical survey variables and additional interaction terms that were significant in separate ANCOVAs

	Sum of squares	*df*	Mean square	*F* ratio	*p*
Corrected model	2656.1	91	29.188	21.599	0.000**
Intercept	11.422	1	11.422	8.452	0.004**
Season	22.926	3	7.642	5.655	0.001**
Time of day	9.745	2	4.872	3.605	0.027*
Adaptation	15.161	3	5.054	3.740	0.011*
Location choice	2.983	3	0.994	0.736	0.531
Health-related mood	1.890	5	0.378	0.280	0.924
Thermal comfort	54.667	3	18.222	13.484	0.000**
Thermal preference	20.167	6	3.361	2.487	0.021*
Shaded or sun-exposed	1.490	1	1.490	1.102	0.294
AC or no AC (5 min ago)	1.192	1	1.192	0.882	0.348
AC or no AC (30 min ago)	0.669	1	0.669	0.495	0.482
Age group	2.350	5	0.470	0.348	0.884
Gender	11.021	1	11.021	8.155	0.004**
Metabolic rate (5 min ago)	11.429	8	1.429	1.057	0.391
Metabolic rate (30 min ago)	6.929	7	0.990	0.732	0.644
Clothing insulation	23.264	14	1.662	1.230	0.247
Globe temperature	29.767	1	29.767	22.027	0.000**
Season * globe temperature	14.789	3	4.930	3.648	0.012*
Thermal comfort * globe temperature	70.231	3	23.410	17.323	0.000**
Time of day * globe temperature	8.257	2	4.129	3.055	0.048*
Thermal preference * globe temperature	13.553	6	2.259	1.671	0.125
Adaptation * shaded or sun-exposed	13.433	3	4.478	3.313	0.019*
Time of day * shaded or sun-exposed	3.567	2	1.189	0.880	0.451
Thermal comfort * shaded or sun-exposed	36.952	3	12.317	9.115	0.000**
Season * shaded or sun-exposed	12.313	3	6.157	4.556	0.011*
Error	1516.253	1122	1.351		
Total	4297.000	1214			
Corrected total	4172.353	1213			

### Neutral temperature, acceptable comfort range, preferred temperature

**Fig. 4:**
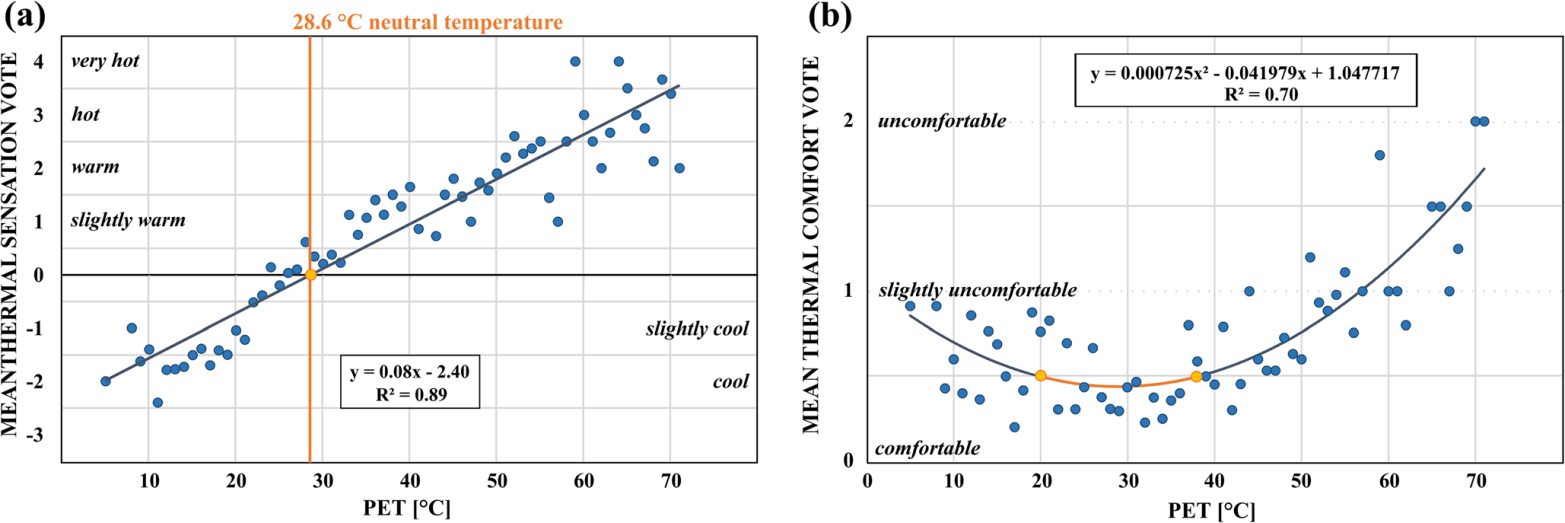
**a** Relationship between mean thermal sensation votes (MTSV) and binned PET (all seasons): linear regression yields a neutral temperature of 28.6 °C. **b** Subjective thermal comfort vs. binned PET (all seasons) reveals an acceptable comfort range of 19.1–38.1 °C

Neutral temperature is the temperature that corresponds to the mean vote of neutral on the thermal sensation scale, i.e., the temperature at which people feel neither cold nor warm, but comfortable (Nikolopoulou and Lykoudis 2006; Lin 2009). To analyze the respondents’ thermal sensitivity to variations in calculated comfort and determine the neutral temperature, we binned PET into 1 °C intervals and calculated the mean thermal sensation vote in each bin. Due to sample size limitations, we calculated neutral temperature for the whole year and not by season. Linear regression revealed a strong relationship between perceived comfort and PET (*R* ^2^ = 0.89, *p* < 0.001), yielding a neutral temperature of 28.6 °C (Fig. 4a). While the neutral temperature found in temperate climates tends to be lower than 20 °C (e.g., 13.3 °C in Sheffield, UK; 18.5 °C in Kassel, Germany), it usually exceeds 22 °C in subtropical and tropical climates, most likely due to adaptation, recent air temperature experience, and expectations (Nikolopoulou and Lykoudis 2006). Spagnolo and de Dear (2003) calculated a neutral temperature of 24.0 °C for subtropical Sydney, Australia. Lin and Matzarakis (2008) found that people at a tourist destination in Taiwan felt comfortable at 27.2 °C. Hwang et al. (2010) determined monthly neutral temperature variations in Taiwan, ranging from 22.3 °C (January) to 28.2 °C (August). Our result falls into the high end of this range, probably because of the dry climate in Tempe. In a more detailed analysis, neutral temperature was not found to vary significantly by adaptation level or short-term thermal history.

To determine the year-round acceptable outdoor thermal comfort range for respondents at the Memorial Union, we used a direct assessment of comfort through the thermal comfort vote. Similar to calculating neutral temperature, thermal comfort votes were averaged for each PET bin and plotted. Using a second-order polynomial curve fit, the curve segment that corresponds to a mean thermal comfort vote of <0.5 represents acceptable thermal comfort conditions (Fig. 4b). Survey results yielded a thermal acceptable range between 19.1 and 38.1 °C. In other climates, upper boundaries of acceptable outdoor conditions were found to be lower, e.g., 21.3–28.5 °C in Taiwan (Lin 2009) and 19–26 °C in Tel Aviv (Cohen et al. 2013).

**Fig. 5:**
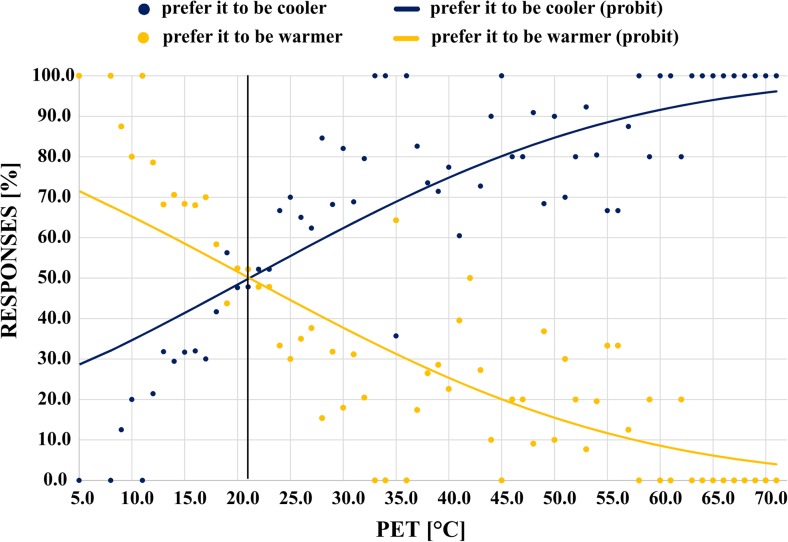
Probit analysis yields 20.8 °C as preferred temperature

The respondents in this study felt comfortable in a wide range of year-round outdoor thermal conditions, but those may not reflect the preferred conditions. We used probit analysis (Ballantyne et al. 1977) to determine the preferred temperature based on reported thermal preferences. The responses were divided into groups that preferred warmer or cooler conditions, creating a binary response variable; neutral responses were split randomly between groups so that probabilities in each temperature bin sum up to 100 % and the transition curves intersect at 50 % level of probability (Spagnolo and de Dear 2003; Yahia and Johansson 2013; Kántor et al. 2016). For each PET interval, we calculated the percent responses in both groups and fitted separate probit curves to the data. The intersection of the two sigmoid curves at 20.8 °C yields the preferred temperature (Fig. 5). In comparison, preferred temperature was found to be higher in tropical and subtropical climates, ranging from 23.0 °C (cold season) and 24.5 °C (hot season) in Taiwan (Lin 2009) to 25.0 °C in Sydney (Spagnolo and de Dear 2003).

### Impact of air-conditioning on thermal stress in the summer

**Fig. 6:**
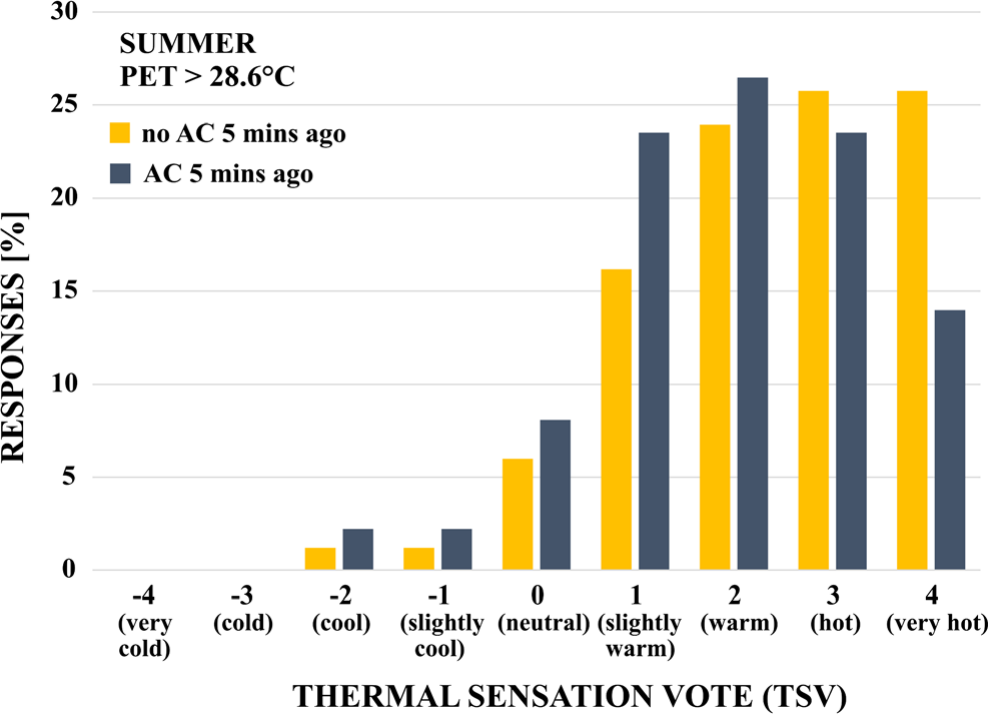
Frequency distribution (in percent) of subjective seasonal thermal sensation votes (TSV) for respondents who were in an air-conditioned space 5 min before the survey vs. respondents who were not; samples from summer survey with conditions above neutral temperature only

The influence of individual short-term thermal history on perceived thermal comfort was addressed by several previous studies (Nikolopoulou and Steemers 2003, Ng and Cheng 2012, Pearlmutter et al. 2014). In our year-round analysis, exposure to AC prior to the survey did not significantly impact reported thermal sensation votes. In a more focused analysis, we investigated subjective comfort in the summer for respondents who were surveyed when conditions were above neutral temperature (>28.6 °C). Figure 6 compares thermal sensation votes for respondents who were exposed to AC 5 min prior to the survey and respondents who were not. An independent samples *t* test showed that subjects with a short-term thermal history of AC exposure reported lower thermal sensation votes (*M* = 1.96, *SD* = 1.38) than those who were exposed to outdoor conditions (*M* = 2.41, SD = 1.35), revealing a statistically significant thermal stress relief for prior exposure to AC, *t*(301) = 2.81, *p* < .005. These findings indicate a lagged thermal response to outdoor conditions, which was already noted by Chen and Ng (2012) in an example of people stepping out of an air-conditioned building seeking sun-exposure even in above neutral thermal conditions. This lag should be investigated further, especially in the context of thermoregulation, short-term and long-term acclimatization, clothing insulation, and individual expectations.

## Discussion and conclusions

Linking field survey responses to meteorological observations, we examined the seasonal impact of shade on outdoor thermal comfort, compared subjective and objective comfort measures, and investigated how various environmental and non-environmental factors impact subjective thermal sensation. We used a Kestrel 4400 Heat Stress Meter to obtain *T*_*mrt*_ from *T*_*g*_. The Kestrel has a 25.4-mm black powder coated copper globe and therefore overestimates *T*_*mrt*_, especially when exposed to the sun, because it absorbs too much short wave radiation (e.g., Kántor and Unger 2011). Due to its small size, convective heat loss increases with higher wind speed, but the response time is significantly reduced compared to standard black globe thermometers (*D* = 150 mm). As wind speed was low when we conducted our field work (0.3 to 1.1 ms^−1^), convective heat loss was minimal. Our case study design limits the validity of our results to calm, clear conditions. The majority of days in Tempe exhibit these conditions, with 80–90 % possible sunshine throughout the year (Table 1, supplemental materials). Furthermore, our results are biased towards a healthy undergraduate student body and should not be generalized to more vulnerable populations, such as the elderly and children.

A regression of binned PET values and mean thermal sensation votes showed that respondents felt neither warm nor cold at 28.6 °C. This neutral temperature was found to be lower in humid and temperate climates. Our analysis of subjective comfort yielded a year-round acceptable outdoor thermal comfort range of 19.1–38.1 °C. Interestingly, the upper boundary of this range corresponds to the “triple digits” Fahrenheit air temperature threshold (38.1 °C = 100.6 °F), which is commonly used by the media and the general public in Arizona to denote the beginning and end of the heat season. While people seem to feel comfortable outdoors in a wide range of conditions, the temperature they prefer is 20.8 °C, as determined by probit analysis. This temperature is representative of air-conditioned environments, indicating that respondents are conditioned to indoor environments, because they are exposed to AC most of the day during the summer. Although short-term exposure to AC was not a significant non-meteorological factor in a year-round thermal comfort analysis (as opposed to adaptation level, gender, thermal comfort vote, thermal preference, season, and time of day), it significantly reduced TSV in the summer when conditions are warmer than neutral temperature. Exposure to AC prior to being outdoors lowered TSV by about half a point on the semantic differential 9-point scale, pointing to a lagged response to heat exposure. These results contribute to the discussion of increased thermal stress for vulnerable populations with no access to AC.

In a seasonal analysis, shade increased thermal comfort significantly in the spring, summer, and fall. Shade reduced TSV by 1 point on the semantic differential 9-point scale, improving subjective thermal sensation from *hot* to *warm* in the summer and from *slightly warm* to *neutral* in the transitional seasons. A multiple regression of TSV on the physical drivers of thermal comfort further emphasized the importance of solar access for thermal sensation. Globe temperature, the integrative measure of air and radiant temperature, was the only statistically significant meteorological predictor of TSV, explaining 51 % of the variation. These findings confirm results from previous studies showing that air temperature alone is not a comprehensive indicator of thermal comfort or stress, because it does not accurately represent the significant variation of thermal conditions in urban environments (e.g., Ali-Toudert and Mayer 2007; Mayer et al. 2008; Lee et al. 2014). Complex shading patterns from buildings and trees modify solar access at the pedestrian level. Therefore, perceived thermal conditions can vary several degrees in the shade and sun, as is evident from the survey respondents’ perceived air temperature estimates. While respondents in direct sun consistently overestimated air temperature, people in the shade underestimated it. Our results show that globe temperature, representative of the radiative environment and solar access, is a prime determinant of thermal comfort and stress in hot dry climates, outperforming indices such as WBGT or the heat index.

Thermal sensation responses did not significantly vary by shade type, suggesting that artificial and natural shade are equally efficient in mitigating heat stress in hot dry climates. Survey results reveal that the human body cannot resolve meteorological differences between shade types when humidity levels are low. This major finding opens up new avenues for active shade management strategies in hot dry climates to mitigate heat stress on citizens. Exposure to extreme heat in desert cities is a hazard of particular concern due to health risks, and it is expected to further increase in the future with projected rapid urbanization and more intense, more frequent, and longer lasting heat waves. Mitigating outdoor thermal stress through photovoltaic canopy shade is especially valuable in dry regions, because photovoltaic structures do not require irrigation and offer the co-benefit of electricity production with high solar potential. Our study did not take into account the esthetics of natural shade, which were found to be significant in recent studies (Klemm et al. 2015); we also did not consider other benefits of trees, such as storm water retention or wildlife habitat. In this context, artificial shade structures cannot replace natural shading, especially in urban green spaces and recreational areas. However, photovoltaic canopies offer a viable shade alternative in desert urban spaces where tree mortality is high or other tree benefits are considered secondary, such as parking lots, bus stops, and pedestrian malls, to create high quality public realm through climate sensitive design.

## Electronic supplementary material

Table 1 Comparison of conditions on field work days to seasonal normals (Phoenix KPHX weather station, 30 year normals (1981-2010), NCDC) (PDF 99.4 kb)

Table 2 Descriptive statistics of the observed variables in each season (PDF 36.5 kb)

Table 3 Multiple regression analysis (*N*=1284) to determine which meteorological observations significantly impact thermal comfort (**p < .01) (PDF 18.3 kb)

Fig. 1 Location of stationary shielded temperature and humidity sensors (2.6 m height) near the Memorial Union. (GIF 7731 kb)

High resolution image (TIFF 56829 kb)

Fig. 2 Daily maximum and minimum air temperature, averaged for shaded and sun-exposed stationary reference sensors; daily mean air temperature and daily mean relative humidity, averaged for all stationary reference sensors; field work days in the summer (June 10, 12, 19, 2014), fall (November 7, 2014), winter (January 22, 2015), and spring (April 2, 2015). (GIF 724 kb)

High resolution image (TIFF 39904 kb)

Fig. 3 Observed globe temperature explains 51 % of the variance in reported thermal sensation votes. (GIF 591 kb)

High resolution image (TIFF 18695 kb)

## References

[CR1] Ali-Toudert F, Mayer H (2007). Effects of asymmetry, galleries, overhanging façades and vegetation on thermal comfort in urban street canyons. Sol Energy.

[CR2] Ballantyne ER, Hill RK, Spencer JW (1977). Probit analysis of thermal sensation assessments. Int J Biometeorol.

[CR3] Chen L, Ng E (2012). Outdoor thermal comfort and outdoor activities: a review of research in the past decade. Cities.

[CR4] Cohen P, Potchter O, Matzarakis A (2013). Human thermal perception of Coastal Mediterranean outdoor urban environments. Appl Geogr.

[CR5] Eliasson I, Knez I, Westerberg U, Thorsson S, Lindberg F (2007). Climate and behaviour in a Nordic city. Landscape Urban Plan.

[CR6] Erell E, Pearlmutter D, Williamson T (2012). Urban microclimate: designing the spaces between buildings.

[CR7] Holst J, Mayer H (2011). Impacts of street design parameters on human-biometeorological variables. Meteorol Z.

[CR8] Höppe P (1999). The physiological equivalent temperature—a universal index for the biometeorological assessment of the thermal environment. Int J Biometeorol.

[CR9] Hwang R-L, Lin T-P, Cheng M-J, Lo J-H (2010). Adaptive comfort model for tree-shaded outdoors in Taiwan. Build Environ.

[CR10] Hwang R-L, Lin T-P, Matzarakis A (2011). Seasonal effects of urban street shading on long-term outdoor thermal comfort. Build Environ.

[CR11] ISO 10551 (1995) Ergonomics of the thermal environment—assessment of the influence of the thermal environment using subjective judgment scales, International Standard, 1st edn. International Organization for Standardization (ISO), Geneva

[CR12] ISO 7726 (1998) Ergonomics of the thermal environment—instruments for measuring physical quantities. International Standard, 2nd edn. International Organization for Standardization (ISO), Geneva

[CR13] ISO 8996 (2004) Ergonomics of the thermal environment—determination of metabolic rate. International Organization for Standardization (ISO), Geneva

[CR14] ISO 9920 (2007) Ergonomics of the thermal environment—estimation of the thermal insulation and water vapour resistance of a clothing ensemble. International Standard, 2nd edn. International Organization for Standardization (ISO), Geneva

[CR15] Johansson E, Emmanuel R (2006). The influence of urban design on outdoor thermal comfort in the hot, humid city of Colombo, Sri Lanka. Int J Biometeorol.

[CR16] Johansson E, Thorsson S, Emmanuel R, Krüger E (2014). Instruments and methods in outdoor thermal comfort studies—the need for standardization. Urban Climate 10. Part.

[CR17] Kántor N, Unger J (2011). The most problematic variable in the course of human-biometeorological comfort assessment—the mean radiant temperature. Cent Eur J Geosci.

[CR18] Kántor N, Égerházi L, Unger J (2012). Subjective estimation of thermal environment in recreational urban spaces—part 1: investigations in Szeged, Hungary. Int J Biometeorol.

[CR19] Kántor N, Kovács A, Takács Á (2016). Seasonal differences in the subjective assessment of outdoor thermal conditions and the impact of analysis techniques on the obtained results. Int J Biometeorol.

[CR20] Klemm W, Heusinkveld BG, Lenzholzer S, Bv H (2015). Street greenery and its physical and psychological impact on thermal comfort. Landscape Urban Plan.

[CR21] Krüger E, Drach P, Emmanuel R, Corbella O (2013). Urban heat island and differences in outdoor comfort levels in Glasgow, UK. Theor Appl Climatol.

[CR22] Lee H, Holst J, Mayer H (2013). Modification of human-biometeorologically significant radiant flux densities by shading as local method to mitigate heat stress in summer within urban street canyons. Adv Meteorol.

[CR23] Lee H, Mayer H, Schindler D (2014). Importance of 3-D radiant flux densities for outdoor human thermal comfort on clear-sky summer days in Freiburg, Southwest Germany. Meteorol Z.

[CR24] Lee H, Mayer H, Chen L (2016). Contribution of trees and grasslands to the mitigation of human heat stress in a residential district of Freiburg, Southwest Germany. Landscape Urban Plan.

[CR25] Lin T-P (2009). Thermal perception, adaptation and attendance in a public square in hot and humid regions. Build Environ.

[CR26] Lin T-P, Matzarakis A (2008). Tourism climate and thermal comfort in Sun Moon Lake, Taiwan. Int J Biometeorol.

[CR27] Lin T-P, Matzarakis A, Hwang R-L (2010). Shading effect on long-term outdoor thermal comfort. Build Environ.

[CR28] Makaremi N, Salleh E, Jaafar MZ, GhaffarianHoseini A (2012). Thermal comfort conditions of shaded outdoor spaces in hot and humid climate of Malaysia. Build Environ.

[CR29] Matzarakis A, Rutz F, Mayer H (2007). Modelling radiation fluxes in simple and complex environments—application of the RayMan model. Int J Biometeorol.

[CR30] Matzarakis A, Rutz F, Mayer H (2010). Modelling radiation fluxes in simple and complex environments: basics of the RayMan model. Int J Biometeorol.

[CR31] Mayer H, Höppe P (1987). Thermal comfort of man in different urban environments. Theor Appl Climatol.

[CR32] Mayer H, Holst J, Dostal P, Imbery F, Schindler D (2008). Human thermal comfort in summer within an urban street canyon in Central Europe. Meteorol Z.

[CR33] Middel A, Häb K, Brazel AJ, Martin C, Guhathakurta S (2014). Impact of urban form and design on microclimate in Phoenix. AZ Landscape Urban Plan.

[CR34] Ng E, Cheng V (2012). Urban human thermal comfort in hot and humid Hong Kong. Energ Buildings.

[CR35] Nikolopoulou M, Lykoudis S (2006). Thermal comfort in outdoor urban spaces: analysis across different European countries. Build Environ.

[CR36] Nikolopoulou M, Steemers K (2003). Thermal comfort and psychological adaptation as a guide for designing urban spaces. Energ Buildings.

[CR37] Nikolopoulou M, Baker N, Steemers K (2001). Thermal comfort in outdoor urban spaces: understanding the human parameter. Sol Energy.

[CR38] Pantavou K, Theoharatos G, Santamouris M, Asimakopoulos D (2013). Outdoor thermal sensation of pedestrians in a Mediterranean climate and a comparison with UTCI. Build Environ.

[CR39] Pearlmutter D, Berliner P, Shaviv E (2007). Urban climatology in arid regions: current research in the Negev desert. Int J Climatol.

[CR40] Pearlmutter D, Jiao D, Garb Y (2014). The relationship between bioclimatic thermal stress and subjective thermal sensation in pedestrian spaces. Int J Biometeorol.

[CR41] Rutty M, Scott D (2015). Bioclimatic comfort and the thermal perceptions and preferences of beach tourists. Int J Biometeorol.

[CR42] Spagnolo J, de Dear R (2003). A field study of thermal comfort in outdoor and semi-outdoor environments in subtropical Sydney Australia. Build Environ.

[CR43] Stewart ID, Oke TR (2012). Local climate zones for urban temperature studies. B Am Meteorol Soc.

[CR44] Thorsson S, Lindqvist M, Lindqvist S (2004). Thermal bioclimatic conditions and patterns of behaviour in an urban park in Göteborg, Sweden. Int J Biometeorol.

[CR45] Thorsson S, Lindberg F, Eliasson I, Holmer B (2007). Different methods for estimating the mean radiant temperature in an outdoor urban setting. Int J Climatol.

[CR46] U.S. Census Bureau (2015) State and County QuickFacts Retrieved from http://quickfacts.census.gov/qfd/states/04/0473000.html

[CR47] Vanos JK, Warland JS, Gillespie TJ, Kenny NA (2010). Review of the physiology of human thermal comfort while exercising in urban landscapes and implications for bioclimatic design. Int J Biometeorol.

[CR48] Vanos JK, Middel A, McKercher GR, Kuras ER, Ruddell BL (2016). A multiscale surface temperature analysis of urban playgrounds in a hot, dry city. Landscape Urban Plan.

[CR49] Western Regional Climate Center (2015) NCDC 1981–2010 Normals for Tempe ASU, Arizona (028499). Retrieved from http://www.wrcc.dri.edu/cgi-bin/cliMAIN.pl?az8499

[CR50] Yahia M, Johansson E (2013). Evaluating the behaviour of different thermal indices by investigating various outdoor urban environments in the hot dry city of Damascus, Syria. Int J Biometeorol.

[CR51] Yin J, Zheng Y, Wu R, Tan J, Ye D, Wang W (2012). An analysis of influential factors on outdoor thermal comfort in summer. Int J Biometeorol.

