# Supplementary material for: Impact of shade on outdoor thermal comfort—a seasonal field study in Tempe, Arizona
Source: Int J Biometeorol. 2016 May 18;60(12):1849–61. doi: 10.1007/s00484-016-1172-5 (PMC5127889; doi:10.1007/s00484-016-1172-5)
Supplement: Supplementary file 1 — Comparison of conditions on field work days to seasonal normals (Phoenix KPHX weather station, 30 year normals (1981-2010), NCDC) (PDF 99.4 kb) [file 484_2016_1172_MOESM1_ESM.pdf]

Field work days	22-Jan-15	2-Apr-15	10-Jun-14	12-Jun-14	19-Jun-14	7-Nov-14
**Mean temperature [°C]**	14.0	24.0	36.0	34.0	32.0	22.0
**Max temperature [°C]**	18.0	31.0	43.0	42.0	39.0	29.0
**Wind velocity [ms^{-1}]**	4.6	4.1	3.9	3.2	2.6	2.2
**Seasonal normals**	**DJF**	**MAM**	**JJA**	**JJA**	**JJA**	**SON**
**Normal daily mean temperature [°C]**	14.0	23.0	33.9	33.9	33.9	24.7
**Normal daily max temperature [°C]**	20.0	29.8	40.4	40.4	40.4	31.1
**Normal mean wind velocity [ms^{-1}]**	2.4	3.0	3.0	3.0	3.0	2.7
**Percent possible sunshine [%]**	79.3	89.3	89.0	89.0	89.0	86.7
**Difference from seasonal normals**						
**Mean temperature [°C]**	0.0	1.0	2.1	0.1	-1.9	-2.7
**Max temperature [°C]**	-2.0	1.2	2.6	1.6	-1.4	-2.1
**Wind velocity [ms^{-1}]**	2.2	1.1	0.9	0.2	-0.4	-0.5

Table 1: Comparison of conditions on field work days to seasonal normals (Phoenix KPHX weather station, 30 year normals (1981-2010), NCDC).

		Surface Temperature	WBGT	Globe Temperature	Air Temperature	Relative Humidity	Dew Point	Heat Index	Wind Speed	K_up	K_down	Ne
		[°C]	[°C]	[°C]	[°C]	[%]	[°C]	[°C]	[m/s]	[Wm-2]	[Wm-2]	[W
**22-Jan-15**	AVE	16.5	10.4	18.8	15.6	15.3	-9.6	12.7	1.1	33.7	129.9	
	SD	5.9	2.4	5.5	2.3	3.6	1.3	1.9	1.4	39.3	192.5	
	MIN	3.0	6.4	11.1	10.8	12.4	-12.2	9.1	0.0	0.0	0.0	
	MAX	35.0	18.7	32.8	19.3	26.2	-6.5	18.0	2.4	134.2	610.0	
**2-Apr-15**	AVE	27.9	19.2	30.5	27.3	16.0	0.6	24.8	0.5	35.2	161.6	
	SD	6.7	2.5	5.1	3.0	3.8	2.5	2.7	0.3	42.3	238.7	
	MIN	13.0	14.2	21.9	20.5	12.2	-4.0	18.4	0.0	0.0	0.0	
	MAX	47.0	25.1	43.3	30.6	25.3	6.3	30.0	1.4	150.4	849.0	
**June 10, 12, 19, 2014**	AVE	38.9	24.4	37.5	34.8	16.6	5.6	35.7	0.6	70.8	284.7	
	SD	8.5	2.6	5.7	3.1	3.5	2.3	4.3	0.4	55.2	343.2	
	MIN	20.0	19.9	29.2	28.3	11.0	2.2	25.8	0.0	26.5	25.2	
	MAX	64.0	31.8	51.7	43.0	33.3	13.0	44.3	1.9	195.0	972.0	
**7-Nov-14**	AVE	22.9	18.2	26.8	24.3	20.0	4.7	22.4	0.3	27.3	99.0	
	SD	5.8	2.8	6.1	3.1	5.8	1.1	2.8	0.4	40.6	175.7	
	MIN	12.0	13.9	18.6	18.2	18.7	2.3	16.6	0.0	0.0	0.0	
	MAX	39.0	27.0	44.7	30.8	40.3	8.1	29.9	2.0	133.6	641.4	

Table 2: Descriptive statistics of the observed variables in each season

	β	*t*	*p*
**(Constant)**		-25.784	0.000**
**WBGT**	0.301	1.750	0.080
**Air Temperature**	0.212	1.432	0.152
**Water Vapor Pressure**	-0.059	-1.159	0.247
**Surface Temperature**	-0.069	-1.052	0.293
**K↓**	-0.154	-1.843	0.066
**K↑**	0.107	1.116	0.265
**Globe temperature**	0.356	2.906	0.004**

Table 3: Multiple regression analysis (*N=1284*) to determine which meteorological observations significantly impact thermal comfort (**$p < .01$).
