# Supplementary material for: Impact of shade on outdoor thermal comfort—a seasonal field study in Tempe, Arizona
Source: Int J Biometeorol. 2016 May 18;60(12):1849–61. doi: 10.1007/s00484-016-1172-5 (PMC5127889; doi:10.1007/s00484-016-1172-5)
Supplement: Supplementary file 2 — Descriptive statistics of the observed variables in each season (PDF 36.5 kb) [file 484_2016_1172_MOESM2_ESM.pdf]

		Surface temperature	WBGT	Globe temperature	Air temperature	Relative humidity	Dew point	Heat index	Wind speed	K_up	K_down	Net radiation
		[°C]	[°C]	[°C]	[°C]	[%]	[°C]	[°C]	[ms^{-1}]	[Wm^{-2}]	[Wm^{-2}]	[Wm^{-2}]
**22-Jan-15**	**AVE**	16.5	10.4	18.8	15.6	15.3	-9.6	12.7	1.1	33.7	129.9	75.5
	**SD**	5.9	2.4	5.5	2.3	3.6	1.3	1.9	1.4	39.3	192.5	149.6
	**MIN**	3.0	6.4	11.1	10.8	12.4	-12.2	9.1	0.0	0.0	0.0	-132.3
	**MAX**	35.0	18.7	32.8	19.3	26.2	-6.5	18.0	2.4	134.2	610.0	491.2
**2-Apr-15**	**AVE**	27.9	19.2	30.5	27.3	16.0	0.6	24.8	0.5	35.2	161.6	111.9
	**SD**	6.7	2.5	5.1	3.0	3.8	2.5	2.7	0.3	42.3	238.7	164.9
	**MIN**	13.0	14.2	21.9	20.5	12.2	-4.0	18.4	0.0	0.0	0.0	-96.8
	**MAX**	47.0	25.1	43.3	30.6	25.3	6.3	30.0	1.4	150.4	849.0	561.6
**June 10, 12, 19, 2014**	**AVE**	38.9	24.4	37.5	34.8	16.6	5.6	35.7	0.6	70.8	284.7	167.5
	**SD**	8.5	2.6	5.7	3.1	3.5	2.3	4.3	0.4	55.2	343.2	215.0
	**MIN**	20.0	19.9	29.2	28.3	11.0	2.2	25.8	0.0	26.5	25.2	-19.8
	**MAX**	64.0	31.8	51.7	43.0	33.3	13.0	44.3	1.9	195.0	972.0	642.7
**7-Nov-14**	**AVE**	22.9	18.2	26.8	24.3	20.0	4.7	22.4	0.3	27.3	99.0	74.5
	**SD**	5.8	2.8	6.1	3.1	5.8	1.1	2.8	0.4	40.6	175.7	162.1
	**MIN**	12.0	13.9	18.6	18.2	18.7	2.3	16.6	0.0	0.0	0.0	-85.6
	**MAX**	39.0	27.0	44.7	30.8	40.3	8.1	29.9	2.0	133.6	641.4	553.1

Table 2: Descriptive statistics of the observed variables in each season
