# Supplementary material for: Impact of shade on outdoor thermal comfort—a seasonal field study in Tempe, Arizona
Source: Int J Biometeorol. 2016 May 18;60(12):1849–61. doi: 10.1007/s00484-016-1172-5 (PMC5127889; doi:10.1007/s00484-016-1172-5)
Supplement: Supplementary file 3 — Multiple regression analysis (N=1284) to determine which meteorological observations significantly impact thermal comfort (**p < .01) (PDF 18.3 kb) [file 484_2016_1172_MOESM3_ESM.pdf]

	β	t	p
**(Constant)**		-25.784	0.000**
**WBGT**	0.301	1.750	0.080
**Air temperature**	0.212	1.432	0.152
**Water vapor pressure**	-0.059	-1.159	0.247
**Surface temperature**	-0.069	-1.052	0.293
**K↓**	-0.154	-1.843	0.066
**K↑**	0.107	1.116	0.265
**Globe temperature**	0.356	2.906	0.004**

Table 3: Multiple regression analysis ($N=1284$) to determine which meteorological observations significantly impact thermal comfort (**$p < .01$).
