# Supplementary figures and images for: Impact of shade on outdoor thermal comfort—a seasonal field study in Tempe, Arizona
Source: Int J Biometeorol. 2016 May 18;60(12):1849–61. doi: 10.1007/s00484-016-1172-5 (PMC5127889; doi:10.1007/s00484-016-1172-5)

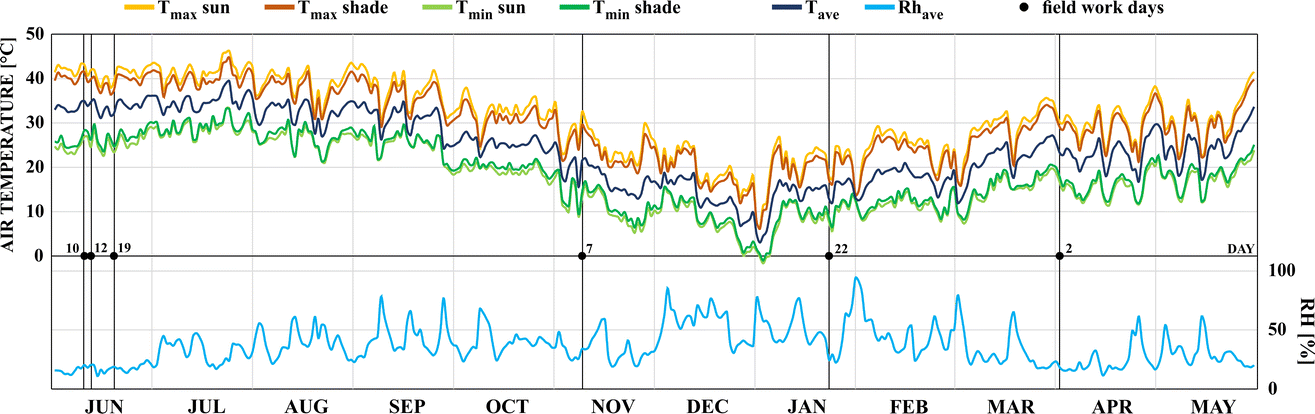

Supplement: Supplementary file 4 — Location of stationary shielded temperature and humidity sensors (2.6 m height) near the Memorial Union. (GIF 7731 kb) [file 484_2016_1172_Fig7_ESM.gif]

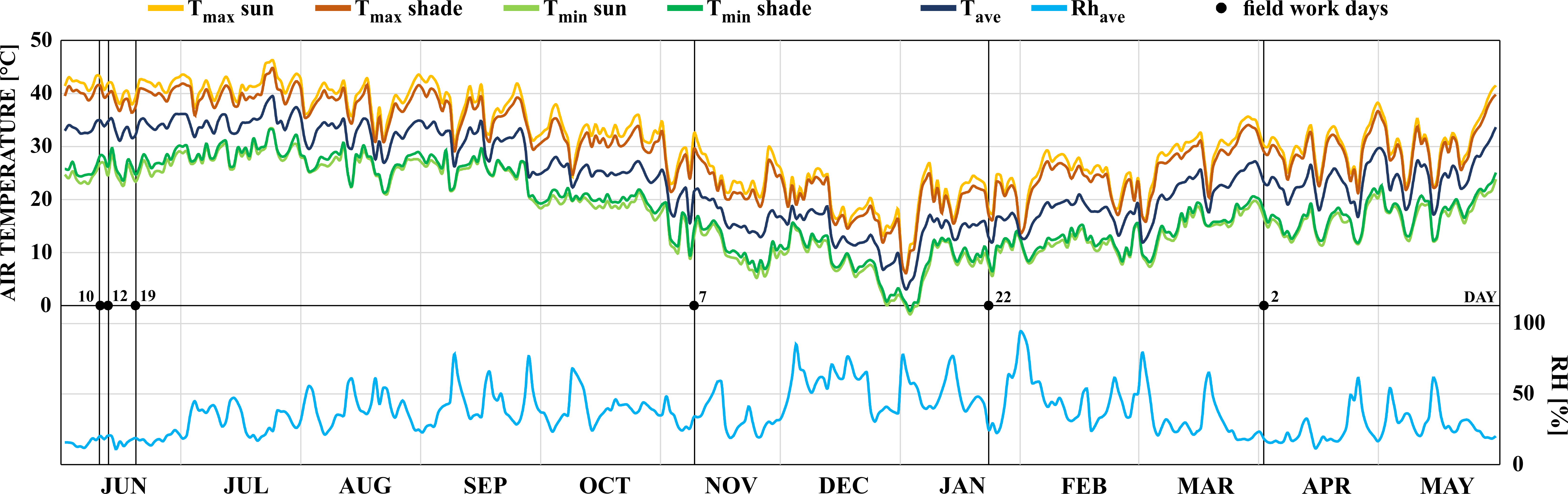

Supplement: Supplementary file 5 — High resolution image (TIFF 56829 kb) [file 484_2016_1172_MOESM4_ESM.tif]

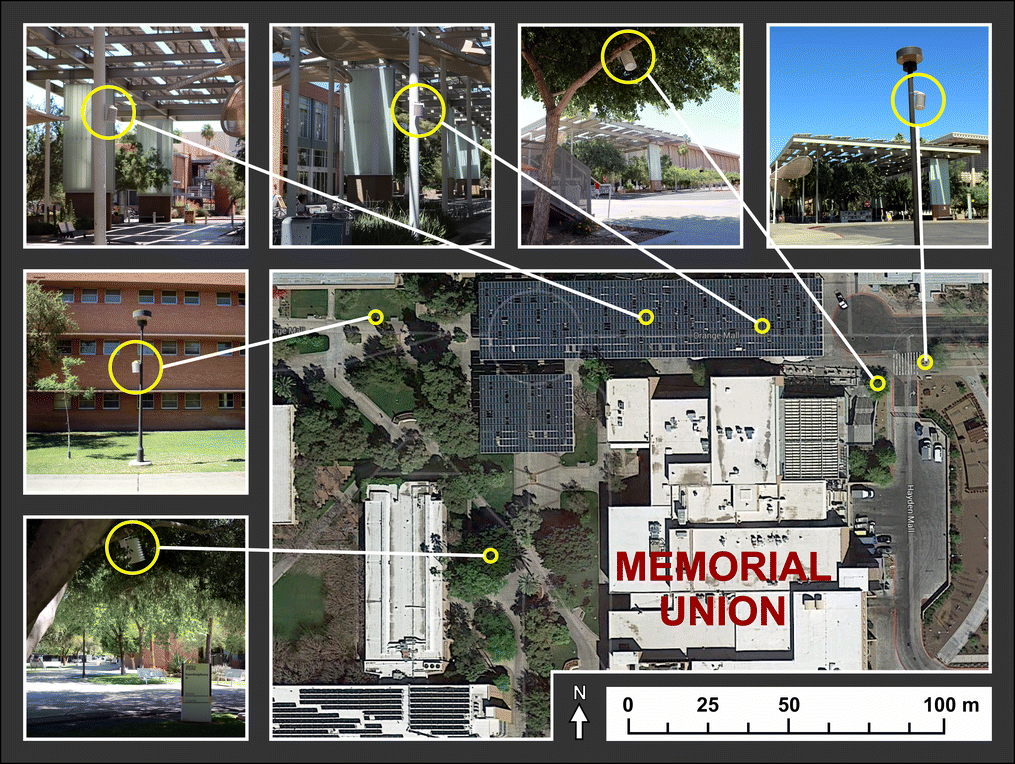

Supplement: Supplementary file 6 — Daily maximum and minimum air temperature, averaged for shaded and sun-exposed stationary reference sensors; daily mean air temperature and daily mean relative humidity, averaged for all stationary reference sensors; field work days in the summer (June 10, 12, 19, 2014), fall (November 7, 2014), winter (January 22, 2015), and spring (April 2, 2015). (GIF 724 kb) [file 484_2016_1172_Fig8_ESM.gif]

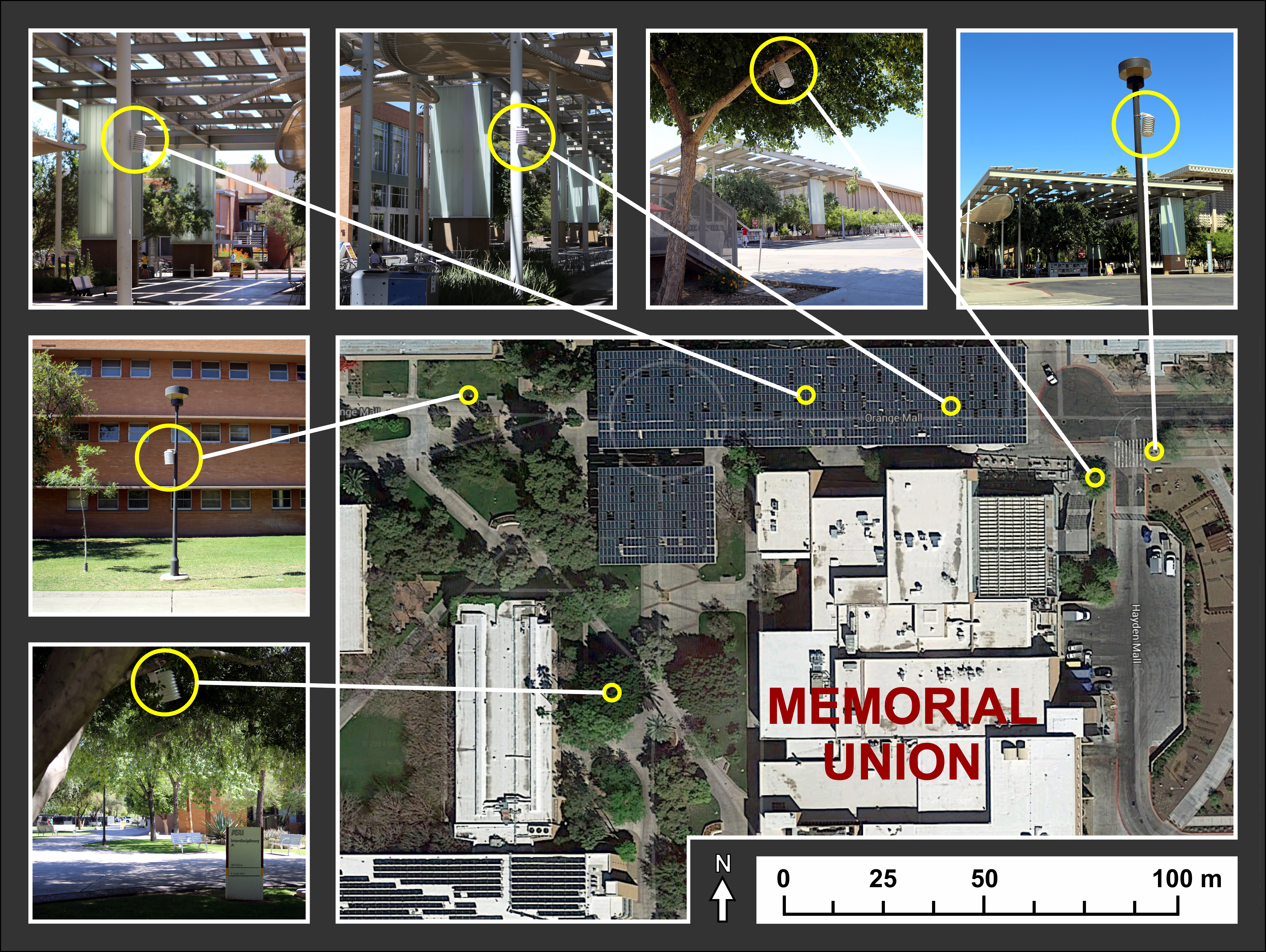

Supplement: Supplementary file 7 — High resolution image (TIFF 39904 kb) [file 484_2016_1172_MOESM5_ESM.tif]

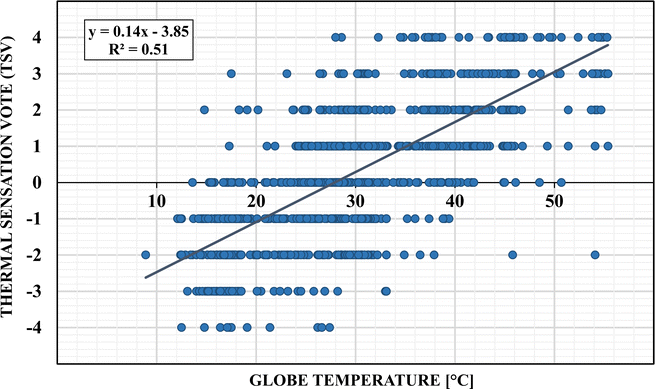

Supplement: Supplementary file 8 — Observed globe temperature explains 51 % of the variance in reported thermal sensation votes. (GIF 591 kb) [file 484_2016_1172_Fig9_ESM.gif]

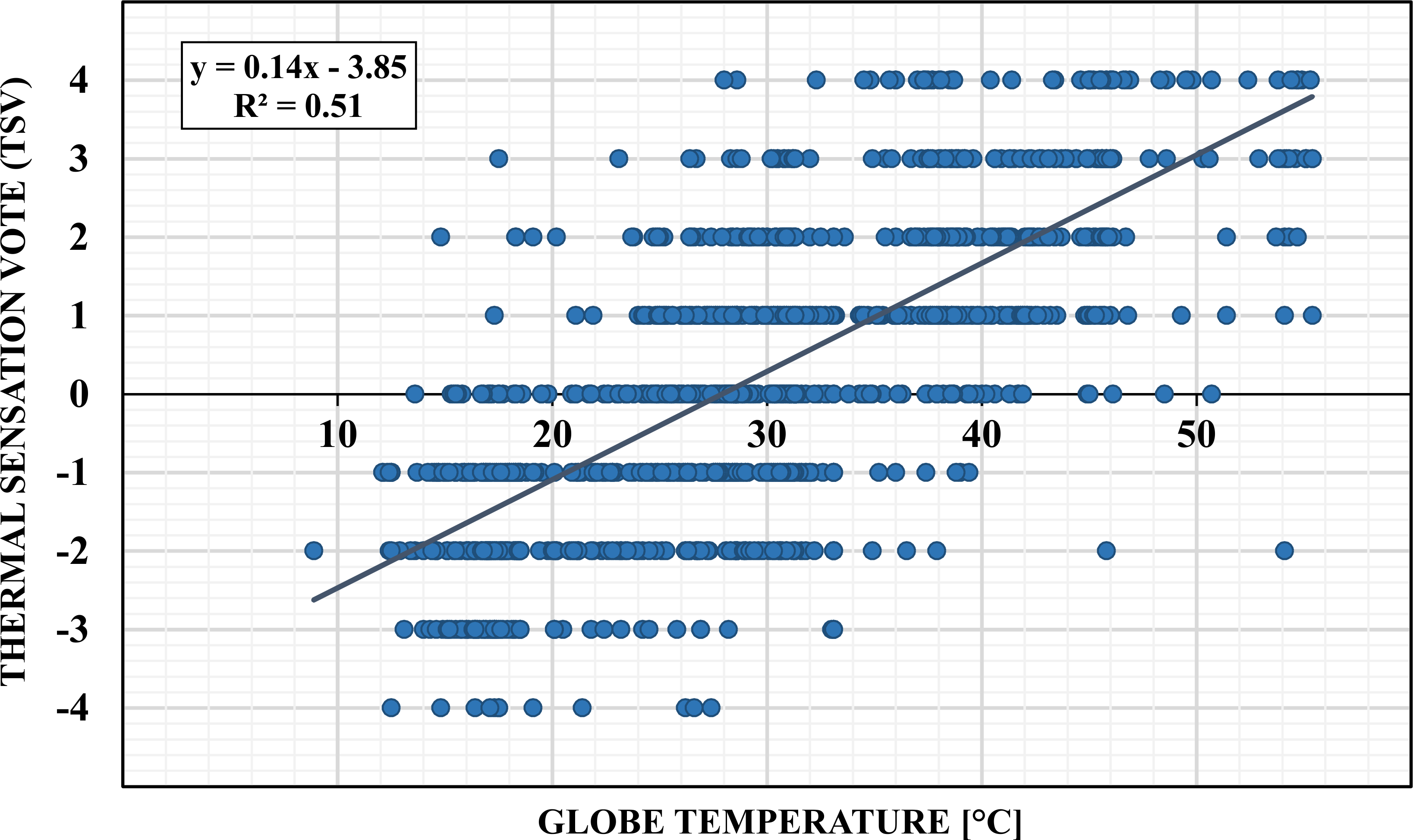

Supplement: Supplementary file 9 — High resolution image (TIFF 18695 kb) [file 484_2016_1172_MOESM6_ESM.tif]
